# Efficacy of an 8-hour education intervention on dementia knowledge, attitude and skills in healthcare professionals in regional hospitals: a nation-wide study from Uganda

**DOI:** 10.11604/pamj.2023.44.165.36470

**Published:** 2023-04-11

**Authors:** Davy Vancampfort, James Mugisha, Samuel Kimbowa, Hafsa Lukwata, Tine Van Damme, Mathieu Vandenbulcke

**Affiliations:** 1 KU Leuven, Department of Rehabilitation Sciences, Leuven, Belgium,; 2 Kyambogo University, Faculty of Social Sciences, Kampala, Uganda,; 3 Butabika National Referral Mental Health Hospital, Kampala, Uganda,; 4 Division of Mental Health, Ministry of Health, Kampala, Uganda,; 5 KU Leuven, Leuven Brain Institute, Department of Neurosciences, Leuven, Belgium

**Keywords:** Competences, dementia, education, low-income country

## Abstract

**Introduction:**

dementia imposes an enormous burden, mainly in low-income countries (LICs). Due to lack of well-trained healthcare professionals, 70-90% of people with dementia do not receive adequate care in LICs. The aim of this study was to evaluate whether a one-day, 8-hour medical education intervention on dementia care improves the knowledge and attitude about and confidence in providing dementia care among healthcare professionals in 8 referral hospitals in Uganda

**Methods:** in this pre-test/post-test study without a control group, participants completed the Alzheimer´s Disease Knowledge Scale (ADKS), Dementia Care Attitude Scale (DCAS), and 9 visual analogue scales (VAS) regarding confidence in specific dementia care skills pre- and post-medical education intervention.

**Results:**

in one hundred twelve healthcare professionals (age = 41.7±10.2 years; 54.5% women), the ADKS, DCAS, and VAS scores for recognizing and assessing core dementia symptoms, communicating effectively, providing psycho-education, activating patients mentally and physically, managing behavioral and psychological symptoms, and involving carers in the treatment improved significantly (P < 0.001) post-medical education intervention.

**Conclusion:**

our study demonstrates that brief educational interventions are efficacious in strengthening the dementia literacy among healthcare professionals in a low-income country. Future research should explore whether such brief educational interventions also result in implementation of efficacious dementia care into routine clinical practice and whether it ultimately may lead to improved health outcomes in patients and formal and informal caregivers.

## Introduction

In the decades to come, Alzheimer´s disease and other dementias will impose an enormous burden on health systems throughout low- and middle-income countries (LMICs), but mainly in sub-Saharan Africa (SSA), as the population in this part of the world is ageing rapidly due to communicable disease mortality decline [1]. Approximately two-thirds of all people with dementia currently live in LMICs and this proportion is expected to rise to more than 70% by 2050 [2]. Dementia is the most important and independent cause of disability and premature mortality in older people living in LMICs [3]. Moreover, it is projected that the burden of serious health-related suffering will, on a global scale, double by 2060, but largest increases will occur in older people with dementia in low-income countries [4].

Despite this high and increasing burden, 70 to 90% of older people with dementia in low-income countries do not receive adequate care [5]. Much of the care for persons with dementia in these countries is provided by family members [6] and family caregivers of people with dementia experience a high caregiving burden [7]. The lack of access to appropriate care for dementia in LMICs should therefore be a health care priority for policy makers and budget holders, in particular since there is evidence available demonstrating that it is possible to provide cost-effective psychosocial interventions for people with dementia in this part of the world [8,9].

In a low-income SSA country as Uganda, where dementia is already perceived as one of the most important causes for disability in older people [10], reasons for low availability of appropriate care is, besides other systemic barriers, largely due to a lack of well-trained human resources, and the high workload among the existing healthcare force [11,12]. It is therefore not surprising that Ugandan healthcare workers providing care to people with dementia experience high levels of psychological distress [13]. Providing dementia-specific training to healthcare professionals has been identified as a possible way to improve dementia care for patients and reduce distress among healthcare workers in low-income settings [14], also in Uganda [12]. Continuous medical education has been used previously as an opportunity to improve the knowledge and skills of the existing workforce in the health sector in Uganda [15]. Little is however known however about outcomes of courses regarding dementia care knowledge, attitudes, and skills among health staff. Such findings are important to inform policy, education and service provision in low-resourced settings.

The aim of this study was to evaluate whether a one-day, 8-hour medical education intervention on dementia care improves the knowledge on, attitude about and confidence in providing dementia care among healthcare professionals working in 8 regional referral hospitals in Uganda. We hypothesize that a brief medical education intervention on dementia care improves the knowledge on, attitude about and confidence in providing dementia care among healthcare professionals.

## Methods

**Study design:** this is a pre-test/post-test study without a control group.

**Study setting:** data were collected in 8 regional referral hospitals across Uganda.

**Study population:** all healthcare professionals working in the mental health units with at least one year of experience and senior health workers from other somatic units participated. There was no restriction in age and gender distribution.

**Study sampling:** for this pilot study, 8 regional referral hospitals were randomly selected via a sealed envelope procedure out of all 14 regional referral hospitals. As only maximum 15 participants per education session were allowed due to ongoing COVID-19 regulations, the hospital board directors of the randomly selected regional referral hospitals selected participants based on either their experience in working with mental health patients or based on their key role in elderly care on the somatic wards (i.e. convenience recruitment). Inclusivity of all disciplines (i.e. at least one nurse, one social worker, one psychologist or psychotherapist, one medical doctor and one psychiatrist or clinical officer with training in clinical psychiatry) had to be ensured.

**Study variables:** before and one day after the one-day medical education intervention participants completed the Alzheimer´s Disease Knowledge Scale (ADKS) [16] Dementia Care Attitude Scale (DCAS) [17] and 9 visual analogue scales (VAS) regarding confidence in specific dementia care skills (at the same time on both days, i.e. 8 am). Questionnaires were not locally adapted. Validation data refer to international studies and not to data in Uganda.

### Alzheimer´s Disease Knowledge Scale (ADKS)

The ADKS [16] comprises 30 true/false statements about the syndrome that are factually correct or incorrect. It covers seven domains: life impact (three items), risk factors (six items), treatment and management (four items), assessment and diagnosis (four items), caregiving (five items), symptoms (four items), and disease course (four items). Each correct answer is worth 1 point. The ADKS score ranges from 0 to 30, with a higher score indicating a better knowledge of dementia. The ADKS is demonstrated adequate psychometric properties for assessing dementia knowledge among healthcare professionals in general and dementia care staff in particular [18]. Dementia Care Attitude Scale (DCAS) The DCAS [17] comprises 10 items graded on a 5-point Likert-type scale with responses varying from ‘strongly disagree’ to ‘strongly agree’. A score of 1 indicates the most negative attitude while a score of 5 indicates the most positive. Four of ten items are negatively worded. The total score was ranged from 10 to 50. Higher score indicated more positive attitudes. To date, the DCAS has not been validated before.

### Confidence in skills to assess and manage people with dementia

Confidence levels were rated using 100 mm visual analogue scales (VAS), where ‘0 mm’ represented ‘not confident at all’, and ‘100 mm’ represented ‘extremely confident’. Confidence in: (a) recognizing core symptoms of dementia, (b) assessing core symptoms of dementia, (c) communicating effectively with people with dementia, (d) providing psycho-education to people with dementia, (e) activating people with dementia mentally, (f) activating people with dementia physically, (g) managing behavioral symptoms, (g) managing psychological symptoms, and (h) involving careers in the treatment of people with dementia were assessed pre- and post-intervention. The psychometric properties of these VAS were not investigated in the present study, although it has been used in prior training interventions to assess confidence in specific, medical skills [19,20]. Prior research demonstrated as well that VAS are valid in assessing confidence for specific medical skills [21]. We considered this VAS approach as the most appropriate for addressing the research aims regarding confidence in dementia care skills, while minimizing participant burden.

### Study intervention

The medical education intervention included 5 hours of interactive, theoretical and 3 hours of practical sessions with role playing exercises and case scenario discussions. A previous review [22] of the most common features of efficacious educational dementia care programs for healthcare workforce indicated that educational dementia care interventions: (a) need to be relevant to participants´ role and experience, (b) involve active face-to-face participation, (c) underpin practice-based learning with theory, (d) are delivered by an experienced facilitator, (e) have a total duration of at least 8 hours, (f) support application of learning in practice, and (g) provide a structured tool or guideline to guide care practice. In developing the educational intervention all these features were considered. The medical education intervention was developed by a local research team consisting of an occupational therapist, a psychologist, a clinical officer with expertise in psychiatry, and a representative of the Ugandan Ministry of Health. All had clinical, research and educational expertise in the field of mental health. Two international experts, one physiotherapist with clinical, research and educational expertise in the field of rehabilitation for people with dementia and one psychiatrist with clinical, research and educational expertise in the field of assessing and managing people with dementia, were consulted. The final content of the 8-hour medical education intervention was discussed with and approved by the Uganda Ministry of Health Mental Health Division. The 8-hour medical education intervention was presented by a psychologist. The psychologist had more than 15 years of expertise in working with people with mental disorders including dementia in the National Mental Health Referral Hospital of Uganda and more than 15 years of academic experience, mainly in community mental health care. As participants were non-experts with no or limited training in dementia care, we relied for the content on dementia care from the Mental Health Gap Action Program Intervention Guide (mhGAP-IG) [23], which was developed by the World Health Organization (WHO) to provide evidence-based guidance for the assessment and integrated management of common mental disorders in non-specialized health settings. The current version of the mhGAP-IG, released in 2016, includes information on essential care, clinical practice, and a master chart of the most common presentations of dementia [23]. The mhGAP-IG intends to contribute towards achieving the specific goals of the WHO´s Comprehensive Mental Health Action Plan 2013-2020, including universal health coverage of mental disorders by providing community-based, comprehensive, integrated, and responsive mental healthcare services. The theoretical part of the training was delivered using tutorials and videos from mhGAP-IG for dementia [23] while the mhGAP-IG for dementia PowerPoint presentations were elaborated with a more prominent focus on the importance of lifestyle psychiatry [24,25]. Practice-based learning was implemented via role playing exercises and discussing case scenarios. Role-plays also have the advantage of established acceptability in mental health training settings in low-income countries [26], and facilitate peer assessment [27]. Manuals were shared with the participants.

**Study procedure:** time to complete the paper-based survey before and one day after the intervention was approximately 30 minutes.

**Statistical analyses:** data were tested for normality with the Shapiro-Wilks test and found to be normally distributed. Participant demographics, ADKS, DCAS and VAS scores are therefore reported as mean ± standard deviation and differences in summed pre- versus post-ADKS, DCAS total scores were examined using Paired student t tests. Within-group effect sizes were calculated using Cohen´s d. The criteria for evaluating the magnitude of the effect size, were small (0.20-0.49), medium (0.50-0.79) and large (≥ 0.80) [28]. The significance level was for all comparisons set at P < 0.0045 after Bonferroni correction (0.05/11). Data were analyzed with SPSS, version 28.

**Ethical considerations:** the study procedure was approved by the ethical committee of the Ugandan National Council of Science and Technology (Reference number SS 4667) and by all local ethical committees of the 8 participating regional referral hospitals (Fort Portal, Gulu, Kabale, Kampala, Masaka, Mbale, Mbarara, Mubende). All participants gave their written informed consent. No financial compensation was provided.

## Results

### Participants

In total, 112 healthcare professionals (age = 41.7 ± 10.2 years; 54.5 % women) participated in the medical education interventions. Most participants were nurses (n=63, 56.2%) of which the majority (n=53, 84.1%) had a specialist mental health degree, followed by social workers (n=8, 7.1%), psychologists or psychotherapists (n=16, 14.2%). medical doctors including general practitioners (n=16, 14.2%), and psychiatrists or clinical officers with training in clinical psychiatry (n=9, 8.0%). In total, 84 (75.0%) was full-time employed. Only 44 (39.3%) of the participants indicated that they had received previously at least to some degree of formal training in dementia assessment and management (e.g. during vocational training, in-service training).

### Changes in knowledge, attitude and skills scores

The ADKS score (19.0±3.0 pre versus 22.8±3.2 post, P < 0.001, Cohen´s d =1.15, 95% confidence interval, CI = 0.91 to 1.39, i.e. large effect) and DCAS (37.3±5.0 pre versus 41.8±4.6 post, P < 0.001, Cohen´s d = 0.93, 95% confidence interval, CI = 0.71 to 1.14, i.e. large effect) scores improved significantly following the 8-hour medical education intervention. With regards to the VAS scores significant improvements were reported for all assessed skills, i.e. VAS scores for recognizing core symptoms of dementia DCAS (53.0±37.2 pre versus 86.3±17.1 post, P < 0.001, Cohen´s d =0.92, 95% confidence interval, CI = 0.71 to 1.15, i.e. large effect), assessing core symptoms of dementia (52.3±37.4 pre versus 85.6±17.1 post, P < 0.001, Cohen´s d = 0.91, 95% confidence interval, CI = 0.74 to 1.19, i.e. large effect), communicating effectively with people with dementia (39.4±36.0 pre versus 82.7±21.7 post, P < 0.001, Cohen´s d =1.11, 95% confidence interval, CI = 0.87 to 1.35, i.e. large effect), providing psycho-education (40.3±37.0 pre versus 83.3±22.0 post, P < 0.001, Cohen´s d =1.08, 95% confidence interval, CI = 0.85 to 1.31, i.e. large effect), activating people with dementia mentally (34.3±30.3 pre versus 75.8±26.6 post, P < 0.001, Cohen´s d = 0.98, 95% confidence interval, CI = 0.75 to 1.20, i.e. large effect), activating people with dementia physically (35.0±33.4 pre versus 75.0±26.6 post, P < 0.001, Cohen´s d = 1.02, 95% confidence interval, CI = 0.79 to 1.24, i.e. large effect), managing behavioral symptoms (37.7±34.4 pre versus 79.1±24.0 post, P < 0.001, Cohen´s d = 1.10, 95% confidence interval, CI = 0.86 to 1.32, i.e. large effect), managing psychological symptoms (36.0±34.4 pre versus 79.5±23.2 post, P < 0.001, Cohen´s d 1.10, 95% confidence interval, CI = 0.86 to 1.32, i.e. large effect), and involving careers in the treatment of people with dementia (47.4±37.0 pre versus 85.7±20.3 post, P < 0.001, Cohen´s d 0.96 =, 95% confidence interval, CI = 0.73 to 1.18, i.e. large effect).

## Discussion

To the best of our knowledge, the current study is the first to demonstrate that an 8-hour medical education intervention improves dementia care knowledge, attitude and perceived skills of healthcare professionals working in regional hospital settings in a low-income country such as Uganda. Our study demonstrates as well that the mhGAP-IG of the WHO could be used as a basis to develop new short medical education programs in low resourced settings. It confirms that it is feasible and efficacious to teach the mhGAP-IG principles to a diverse groups of healthcare professionals (i.e. with different backgrounds and levels of education and expertise) working with elderly in low resourced settings. This underscores the universal aspect of the mhGAP-IG of the WHO, which can be used to build a common understanding in medical education programs and healthcare settings about the assessment and management of mental health disorders such as dementia [29].

More in detail, a first important finding of the current study is that participants indicate that following the medical education intervention they felt more confident in recognizing and assessing symptoms of dementia. Although it is uncertain whether this increased confidence will also result in improved clinical practices, a previous study in rural Uganda demonstrated that healthcare professionals trained in dementia care are using more often than non-trained healthcare professionals neuropsychological tests, blood tests, urine tests and even brain imaging, if available, when assessing patients for dementia [12]. Second, following the medical education intervention, healthcare professionals were more confident in activating people with dementia not only mentally, but also more physically in routine care. This is important as, to date, in LMICs, only cognitive stimulation therapies have been successfully implemented in dementia care [9]. There is however increasing evidence for the beneficial effects of physical activity on quality of life and performance of daily life activities in people with dementia [30].

Moreover, there is clear evidence for the added value of combining cognitive stimulation with physical activity within dementia care [31]. Because physical activity may be implemented at low cost and often requires minimal resources, training and skills on the part of the provider and the individual being active, it may be a feasible complementary treatment option in low-resourced settings [32]. Third, since healthcare professionals reported that following the medical education intervention they felt more confident to engage family caregivers in the treatment, these family caregivers should ideally be involved as well in physically activating people with dementia, also for their own welfare. A recent study in rural, southwestern Uganda demonstrates that, for example, active gardening reduces symptoms of depression, anxiety and stress in family caregivers of people with dementia [33]. Finally, an important observation was that following the medical education intervention healthcare professionals felt more confident in managing psychological and behavioral symptoms. This finding is important as a recent qualitative study [13] indicated that, in particular, lack of confidence in dealing with dementia related problematic behaviors, such as wandering and aggression are important stressors for formal caregivers in Uganda. Future longitudinal research should therefore explore whether training healthcare professionals in low-income countries in coping with these symptoms will also result in lower stress levels, less compassion fatigue and less burnout among healthcare professionals.

### Strengths, limitations and future research

This study has several strengths. It is the first to propose a brief medical education intervention proven to be efficacious in filling the gap to train healthcare professionals on dementia care in remote areas across a low-income country. The short-term nature of the intervention is also a benefit since this will facilitate replication and scale-up. Despite these strengths, the study had also some limitations. First, although our assessment tools have been used before for evaluating education interventions [34,35], the validity in healthcare professionals in LMICs is unknown. Second, we only evaluated the immediate (one day after) impact of the medical education intervention on subjective knowledge, attitude improvements and confidence gain while the long-term effects and the impact on actual clinical practice are unknown. It is recommended that reliable and valid assessment tools will be used in future studies to evaluate, for example, competences pre- and post a medical education intervention in daily clinical practice. To this end, specific evaluation tools have been developed, such as recently The Dementia Care Competence Scale [36,37]. Ideally, long-term (>1 year) trials should investigate whether clinician-targeted medical education interventions lead to health outcome changes in older people with dementia, but also in less stress and burnout among formal and informal caregivers. This would allow for the public health impact of such interventions to be effectively quantified. Finally, future research should explore in more detail which health care professionals benefitted the most from the medical education intervention. Due to the fact that the numbers of psychologists or psychotherapists, medical doctors including general practitioners, and psychiatrists or clinical officers with training were rather limited, we did not explore subgroup analyses.

## Conclusion

It can be concluded that providing healthcare professionals in regional low-resourced settings in a low-income country with dementia care training is able to improve the knowledge, attitude and confidence of the participants. Future research should explore whether brief medical education interventions may also be an important component in effectively implementing efficacious dementia care into routine clinical practice and whether it ultimately may lead to improved health outcomes in patients and informal and formal caregivers in these low-resourced settings.

### What is known about this topic

- *The burden of dementia is worldwide increasing, particularly in low-income countries;*
- *Despite a high and increasing disease burden, 70 to 90% of older people with dementia in low-income countries do not receive adequate care;*
- *Besides the high workload among the existing healthcare force, the most important reason for inadequate dementia care in low-income countries is a lack of well-trained care providers*.

### What this study adds

- *A brief medical education intervention improves dementia care knowledge, attitude and perceived skills in Ugandan healthcare professionals working in regional hospitals;*
- *In a low-income country as Uganda, the Mental Health Gap Action Program Intervention Guide (mhGAP-IG) is a useful source for developing brief medical education interventions;*
